# Supplementary material for: Masking of a circadian behavior in larval zebrafish involves the thalamo-habenula pathway
Source: Sci Rep. 2017 Jun 22;7:4104. doi: 10.1038/s41598-017-04205-7 (PMC5481326; doi:10.1038/s41598-017-04205-7)
Supplement: Supplementary file 3 — Supplementary Information [file 41598_2017_4205_MOESM3_ESM.pdf]

Masking of a circadian behavior in larval zebrafish involves the thalamo-habenula pathway

Qian Lin and Suresh Jesuthasan

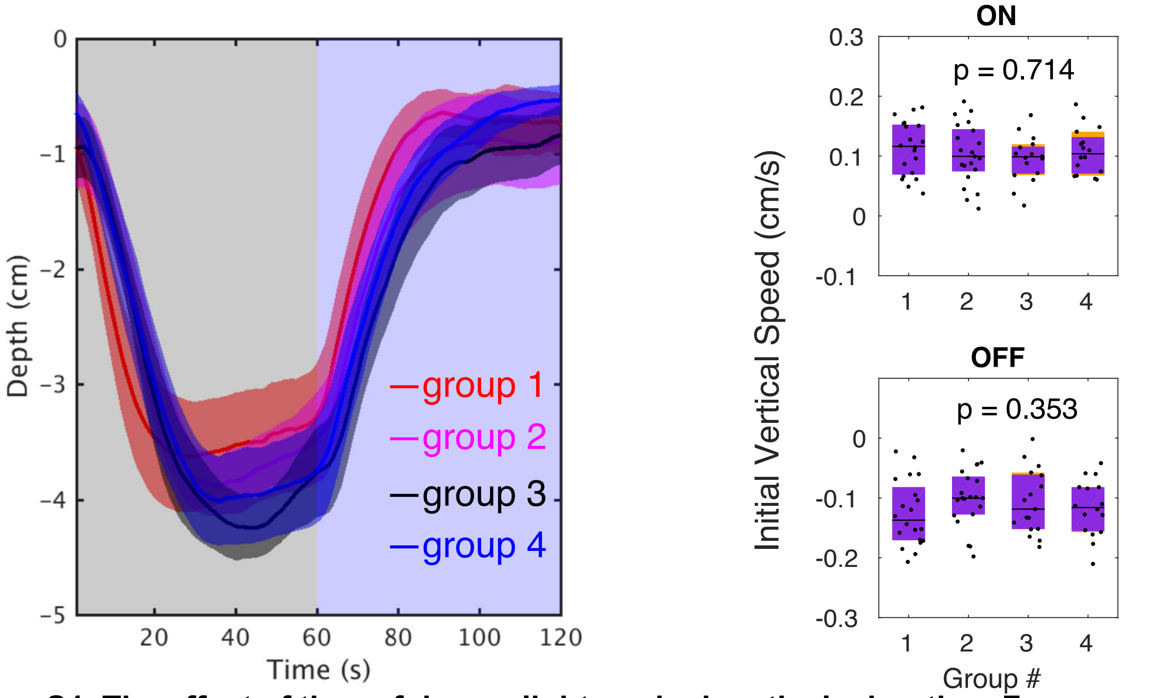

Figure S1. The effect of time of day on light-evoked vertical migration. Four groups of 2-week-old larvae from the same clutch were tested sequentially. Group 1: 11 am – 1 pm, n = 20; group 2: 1pm -3 pm, n = 21; group 3: 3pm -5 pm, n = 17; group 4: 5 pm -7 pm, n = 16. **(a)** Mean position during light OFF and ON. Shadows indicate 95% confidence interval. **(b)** Initial vertical speed. For diving triggered by light offset, χ^2 = 1.36, df = 3, p = 0.353, Kruskal-Wallis test; for climbing upon light onset, χ^2 = 3.26, df = 3, p = 0.714, Kruskal-Wallis test. Black lines indicate the median values and purple patches for the quartiles. There was no significant difference in the vertical position of fish in the last 20 s of the light or dark period, light period: χ^2 = 2.64, df = 3, p = 0.450, Kruskal-Wallis test; dark period, χ^2 = 6.42, df = 3, p = 0.0927, Kruskal-Wallis test.

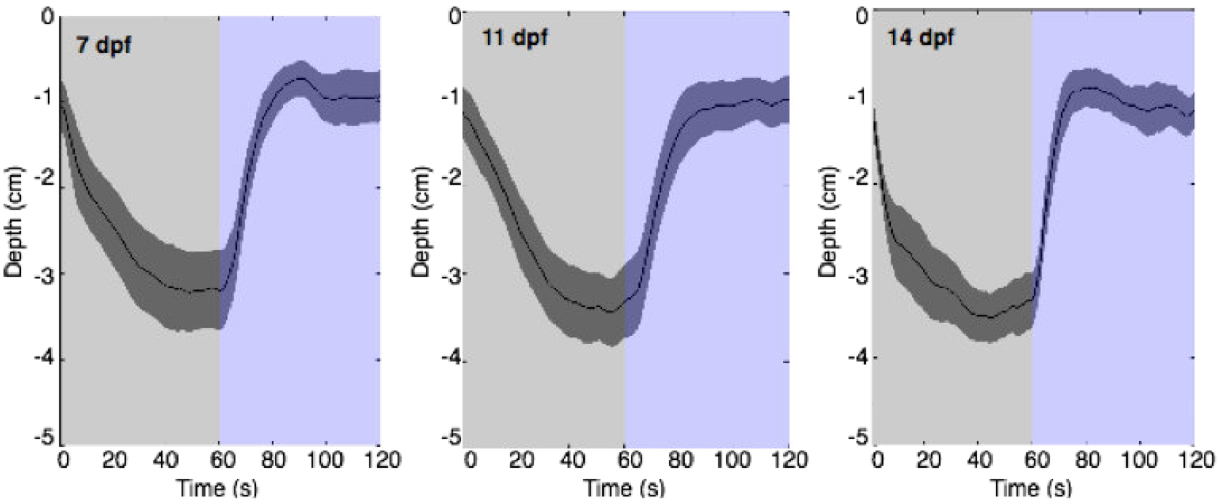

Figure S2. The effect of age on light-evoked vertical migration of zebrafish larvae. Traces show the mean position of fish during light OFF and light ON, at 7 dpf, 11 dpf and 14 dpf. N = 25. Shadows indicate 95% confidence interval.

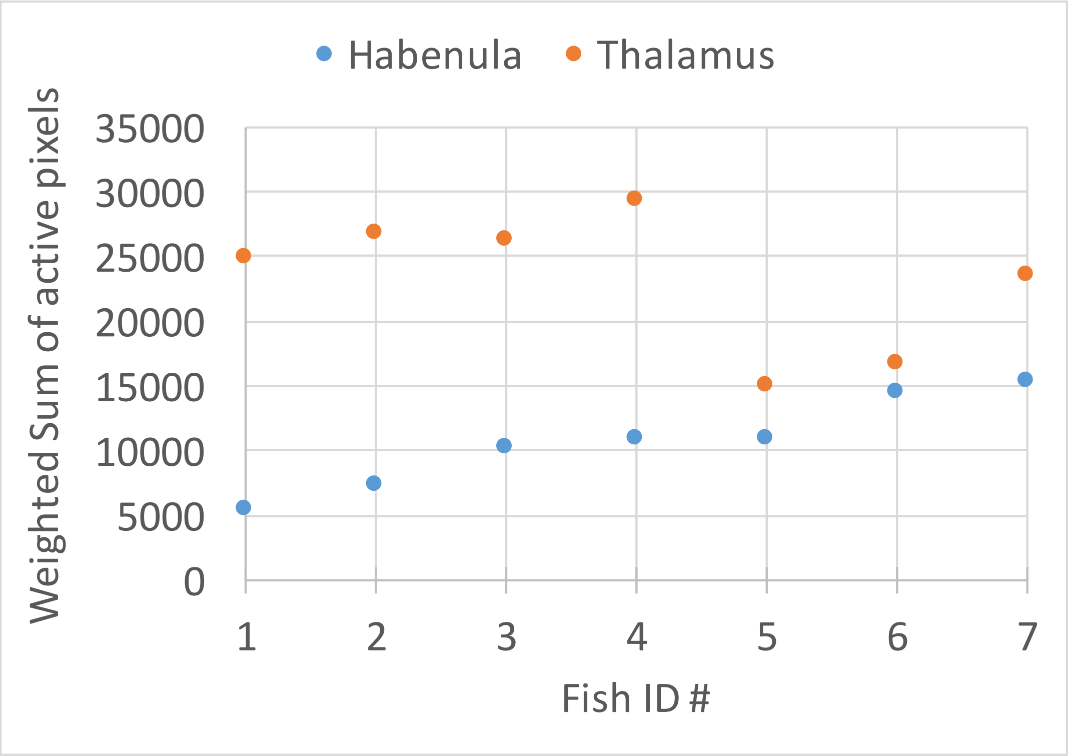

Figure S3. Comparison of tonic response to blue light in the thalamus and habenula of different fish. This chart shows the weighted sum of active pixels in the ICA spatial map. The corresponding ICA temporal signals have r > 0.5 compared to the template.

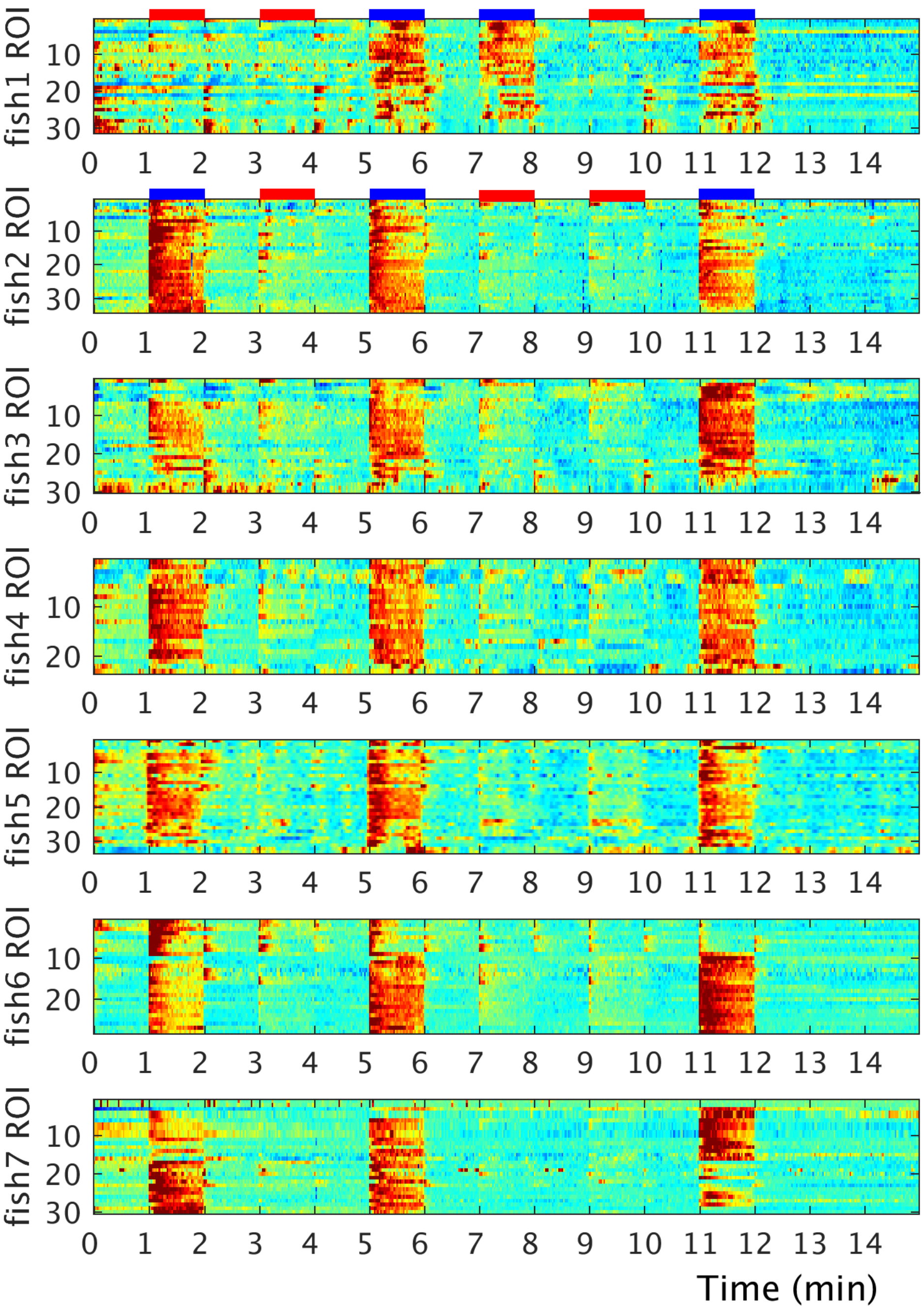

Figure S4. Comparison of neural response in 7 fish, as shown by calcium imaging. Each row indicates the Z score in an ROI with tonic response to blue light. ROIs were derived from ICA spatial maps that were first smoothened by a gaussian kernel, then thresholded by a standard deviation of 2; segments with an area of > 200 pixels were chosen as ROIs (Mukamel et al, 2009).

Legends for videos files

Movie 1. Lesion of the left dorsal neuropil

A z-stack through the habenula after the neuropil of the dorsal left habenula was lesioned. The cavity induced by the lesion is colored magenta, while habenula neurons are visible because of the expression of GCaMP3 under the s1011t GAL4 driver. Planes are 3 μm apart. Anterior is to the top.

Movie 2. Lesion of the ventral neuropils

A z-stack through the habenula after the neuropil of bilateral lesion of the ventral regions of the habenula. The cavity induced by the lesion is colored magenta, while habenula neurons are visible because of the expression of GCaMP3. Planes are 3 μm apart. Anterior is to the top.
